# Midlife Life’s Simple 7, Psychosocial Health, and Physical Frailty, Hospital Frailty, and Comprehensive Frailty 10 Years Later

**DOI:** 10.3390/nu15102412

**Published:** 2023-05-22

**Authors:** Qi Wang, Chunmiao Zhou, Caiyun Dong, Jiajun Zhang, Ziwei Xie, Huizi Sun, Chunying Fu, Wenting Hao, Dongshan Zhu

**Affiliations:** 1 Department of Epidemiology, School of Public Health, Cheeloo College of Medicine, Shandong University, Jinan 250012, China; wangqii2019@outlook.com (Q.W.); z13630813223@163.com (C.Z.); acaiyundong@163.com (C.D.); zoey7gg@163.com (Z.X.); sunhui_0909@126.com (H.S.); fuchunying_z@163.com (C.F.); 2 Centre for Health Management and Policy Research, School of Public Health, Cheeloo College of Medicine, Shandong University, Jinan 250012, China; jjzhang@aging.org.cn (J.Z.); haowenting95@126.com (W.H.); 3 National Health Commission Key Lab of Health Economics and Policy Research, Shandong University, Jinan 250012, China

**Keywords:** Life’s Simple 7, psychosocial health, frailty index, frailty phenotype, hospital frailty

## Abstract

This study aims to examine the associations between midlife Life’s Simple 7 (LS7) status, psychosocial health (social isolation and loneliness), and late-life multidimensional frailty indicators, and to investigate their synergistic effect on frailty. We used cohort data from the UK Biobank. Frailty was assessed using physical frailty phenotype, hospital frailty risk score, and frailty index. Cox proportional-hazards models were used to estimate the hazard ratios (HRs) and 95% confidence intervals (CIs) on the association between the LS7 score, psychosocial health, and frailty. For the association of LS7 with physical and comprehensive frailty, 39,047 individuals were included. After a median follow-up of 9.0 years, 1329 (3.4%) people were identified with physical frailty, and 5699 (14.6%) with comprehensive frailty. For the association of LS7 with hospital frailty, 366,570 people were included. After a median follow-up of 12.0 years, 18,737 (5.1%) people were identified with hospital frailty. Compared to people with a poor LS7 score, those with an intermediate (physical frailty: 0.64, 0.54–0.77; hospital frailty: 0.60, 0.58–0.62; and comprehensive frailty: 0.77, 0.69–0.86) and optimal LS7 score (physical frailty: 0.31, 0.25–0.39; hospital frailty: 0.39, 0.37–0.41; and comprehensive frailty: 0.62, 0.55–0.69) were associated with a lower risk of frailty. Poor psychosocial health was associated with an increased risk of frailty. People who had a poor psychosocial status and poor LS7 score had the highest risk of frailty. A better LS7 score in midlife was associated with a reduced risk of physical, hospital, and comprehensive frailty. There was a synergistic effect of psychosocial status and LS7 on frailty.

## 1. Introduction

Frailty, a complex age-related clinical condition, is characterized by a decline in physiological capacity across multiple organ systems, with a resultant increased vulnerability to stressors [1,2]. Frailty has been associated with an increased risk for poor health outcomes including falls, incident disability, hospitalization, and mortality [3]. Several metrics have been developed to define and assess frailty in various domains, such as the frailty index (FI) for comprehensive frailty, frailty phenotype (FP) for physical frailty, and hospital frailty risk scores (HFRS) [1]. Although frailty prevalence greatly varies across studies (range of 4–59%) [4], due to the lack of standardized concepts or measures, all older adults are at risk of developing frailty. There will be 1.5 billion people aged 65 or older by 2050 [5], which means more people might live with frailty. Given the poor health outcomes related to frailty, it is imperative that we identify modifiable risk factors to delay or prevent the development of frailty.

Ageing, the psychosocial environment, and morbidity conditions act together to drive the development of frailty [1]. Among them, cardiovascular health (CVH) status is closely related to frailty. The American Heart Association (AHA) has introduced Life’s Simple 7 (LS7) metrics to assess and promote CVH. The LS7 includes four modifiable lifestyle behaviors (not smoking, healthy weight, eating healthy, and being physically active) and three biometric measures (blood pressure, cholesterol, and blood sugar) [6]. The existing few studies on LS7 and frailty have focused on the association with FP (i.e., physical frailty) [7,8]. These two studies reported that a higher LS7 score in midlife/the elderly was associated with a reduced risk of physical frailty. However, no study has examined the relationship between LS7 in midlife and FI (comprehensive frailty) or HFRS (hospital frailty) in later life.

Besides the use of the LS7 as metrics for CVH, psychosocial environment factors (e.g., social isolation and loneliness) may also influence the development of frailty. Evidence on the association between social isolation, loneliness, and frailty has been inconsistent [9,10,11]. Moreover, there might be a synergistic effect of psychosocial factors and LS7 on frailty. Thus, based on the bio-psycho-social medical model, we aimed to examine the associations between midlife LS7 status, psychosocial health (social isolation and loneliness), and late-life multidimensional frailty indicators (physical frailty, hospital frailty, and comprehensive frailty), and to investigate their synergistic effect on frailty.

## 2. Materials and Methods

### 2.1. Study Design and Participants

The UK Biobank is a large population-based prospective cohort study that recruited over 500,000 participants aged 40–69 years between 2006 and 2010 [12]. Participants attended 1 of 22 assessment centers across England, Wales, and Scotland, where they completed the touchscreen questionnaire, underwent physiological measurement, and provided biological samples at a baseline assessment visit. Participants gave informed consent for data linkage to national hospital inpatient admissions, cancer registrations, and death registrations. UK Biobank received ethical approval from the UK National Health Service’s National Research Ethics Service (ref 11/NW/0382).

In this study, a prospective design was adopted based on participants with no frailty at baseline. For assessment of physical frailty (using FP) and comprehensive frailty (using FI), participants were followed up from baseline (2006–2010) to Visit 2 (2014–2019). As only part of population at baseline was selected for a follow-up in the UK Biobank, 39,047 individuals were finally included in the association with FP and FI. For hospital frailty (using HFRS) assessment, all people who had health data linkage to primary care or hospital admission records in 2021 were included; 366,570 people were finally included in the association with hospital frailty. This research was conducted under UK Biobank application number 68369.

### 2.2. Exposure

Based on AHA recommendations, the LS7 score (range 0–14) was a sum of 7 metrics; each metric was scored 0 (poor), 1 (intermediate), and 2 (optimal) [6]. A higher score indicated a more optimal CVH. The overall LS7 score was categorized as poor (0 to 5), intermediate (6 to 9), and optimal (10 to 14) as suggested in previous research [13]. The LS7 include four modifiable lifestyle behaviors and three biometric measures. We thus also calculated a lifestyle score (including smoking status, body mass index (BMI), physical activity, and diet) ranging from 0 to 8, and a biometric score (including blood pressure, cholesterol, and glycemic status) ranging from 0 to 6 [14]. The lifestyle score was also categorized as poor (0–2), intermediate (3–5), and optimal (6–8), and the biometric score was categorized as poor (0–1), intermediate (2–3), and optimal (4–6). The UK Biobank fields used in the construction of the LS7 score were shown in Table S1. Definitions of each component of LS7 were summarized in Table S2 [13].

Psychosocial health status was measured by social isolation and loneliness. Social isolation was derived from three questions and each question was scored 0–1 (Table S3). Social isolation status was categorized as ‘with’ (scored 2 or 3) and ‘without’ (scored 0 or 1) [15]. Loneliness was a binary variable, with 0 indicating no feeling of loneliness, and 1 indicating feeling loneliness. The overall psychosocial health status was a sum of social isolation score and loneliness score, and was divided into ‘good’ (not isolated and lonely) and ‘poor’ (isolated, lonely, or both).

### 2.3. Outcome

We used three frailty measures of FP, HFRS, and FI to assess physical frailty, hospital frailty, and comprehensive frailty, respectively.

We used the FP metrics derived by Fried et al. and ever validated by UK Biobank data [16]. FP was defined using five clinical features: unintentional weight loss, exhaustion, low physical activity, slow walking speed, and low grip strength (weakness). Participants were classified as frail if they presented three or more features, pre-frail if they presented one or two features, and robust if they had none of the features [17]. The detailed definitions of each clinical feature of FP were shown in Table S4.

The FI metrics, which had previously been validated using baseline data of UK Biobank [18], included a cumulative count of 48 self-reported health conditions [19]. FI was derived from the total number of conditions presented in an individual divided by the total possible count. It was a value between 0 and 1, and a higher value indicated a greater degree of frailty. Participants were classified as being robust (<0.08), pre-frail (0.08–0.25), or frail (≥0.25) [20]. The detailed items and scoring of FI were described in Table S5.

The HFRS was computed based on the International Classification of Diseases 10th Revision (ICD-10) codes from hospital records. HFRS had also been validated from UK Biobank data [21]. Each of the 109 frailty-related ICD-10 codes was assigned a weight ranging from 0.1 to 7.1, depending on the strength of the association with frailty (Table S6). The HFRS was calculated by summing all the weighted codes, and categorized into low (<5), intermediate (5–15), and high (>15) risk of frailty [22].

We constructed a binary variable for frailty, with robust (low) and pre-frail (intermediate) representing “no frailty”, and frail (high) representing “frailty”. Individuals classified as “no frailty” were used as the reference group.

### 2.4. Covariates

We included the following factors in all analyses as covariates: age, sex, race/ethnicity, education, income level, and alcohol status. Race/ethnicity was categorized as white and non-white. Years of education was categorized as ≤10, 11–12, and >12 years. Income level was divided into four categories: level 1 (Less than £18,000), level 2 (£18,000 to £30,999), level 3 (£31,000 to £51,999), and level 4 (greater than 52,000). Alcohol status was categorized as never drinking, previous drinking, and current drinking. In the association with FP, since no illness factors were contained, we also included the following additional variables: diabetes status, family history of cardiovascular disease (CVD), stroke status, hypertension status, and CVD status. These variables were dichotomized as present or absent based on self-report at baseline.

### 2.5. Statistical Analysis

Baseline characteristics were presented as means and standard deviation (SD) for continuous variables and as percentages (%) for categorical variables. Normality was tested by using Shapiro–Wilk, P–P plots, and Q–Q plots. Differences between groups were compared by using ANOVA (continuous variables) or chi-square test (categorical variables). Cox proportional-hazards regression models were used to estimate the hazard ratios (HR) and 95% confidence intervals (CI) between LS7 score (‘poor’ as the reference group) and psychosocial health (‘good’ as the reference group) and frailty. Each component of LS7 and psychosocial health factor with frailty was also analyzed. For participants who experienced frailty, follow-up time was calculated as their age when frailty was observed minus baseline age; for participants who were not experiencing frailty, follow-up time was defined as their age at the last follow-up (censored date: January 2021 for HFRS, and January 2019 for FI and FP) minus baseline age. All analyses were adjusted for age at the last follow-up, sex, years of education, race/ethnicity, income level, and alcohol status. In the association with FP, diabetes status, family history of CVD, stroke status, hypertension status, and CVD status were further adjusted.

We also analyzed the synergistic effects of LS7 and psychosocial health on different frailty dimensions. To quantify dose–response relationships, we used restricted cubic spline models to test the non linear associations between LS7 score and frailty. All analyses were performed in SAS 9.4 and R (v4.2.1).

## 3. Results

### 3.1. Characteristics of the Study Participants

The characteristics of 39,047 participants for the assessment of physical frailty and comprehensive frailty were shown in Table 1. The mean baseline age was 64.0 (SD 7.6) years, and 19,909 (51.0%) participants were females. During follow-up, 1864 (4.8%) participants had a poor LS7 score, 24,926 (63.8%) had an intermediate LS7 score, and 12,257 (31.4%) had an optimal LS7 score. Only 731 (1.9%) had a poor psychosocial health status. Compared with individuals with a poor or intermediate LS7 score, those with an optimal LS7 score were more likely to be female, younger, white, have a higher education level, have a higher income level, and never alcohol intake. After a median follow-up of 9.0 years, FP identified 1329 (3.4%) people with physical frailty, and FI identified 5699 (14.6%) with comprehensive frailty. The characteristics of 366,570 participants for the assessment of hospital frailty were shown in Table S7. After a median follow-up of 12.0 years, 18,737 (5.1%) people were identified with hospital frailty.

### 3.2. LS7 Score and Physical Frailty, Hospital Frailty, and Comprehensive Frailty

Compared to people with a poor LS7 score, those with an intermediate (physical frailty: HR 0.64, 95%CI 0.54–0.77; comprehensive frailty: 0.77, 0.69–0.86) and optimal LS7 score (physical frailty: 0.31, 0.25–0.39; comprehensive frailty: 0.62, 0.55–0.69) were associated with a lower risk of frailty (Table 2). When the relationship between LS7 score and hospital frailty were analyzed, an intermediate and optimal LS7 score was related to a 40% (0.60, 0.58–0.62) and 60% (0.39, 0.37–0.41) lower risk of hospital frailty, respectively (Table 3). When the associations between lifestyle score, biometric score of LS7, and frailty were analyzed, a higher lifestyle score was linked to a lower risk of physical frailty, comprehensive frailty, and hospital frailty. Specifically, compared with poor BMI, moderate and optimal BMIs were all associated with a lower risk of physical frailty, comprehensive frailty, and hospital frailty. Moreover, an optimal diet was associated with a decreased risk of frailty, with HRs (95%CI) of 0.74 (0.56, 0.98), 0.83 (0.73, 0.95), and 0.69 (0.62, 0.78), respectively (Table 2 and Table 3). No association was found between biometric score and physical frailty or comprehensive frailty, while a higher biometric score was associated with a lower risk of hospital frailty (Table 2 and Table 3).

**Figure 1:**
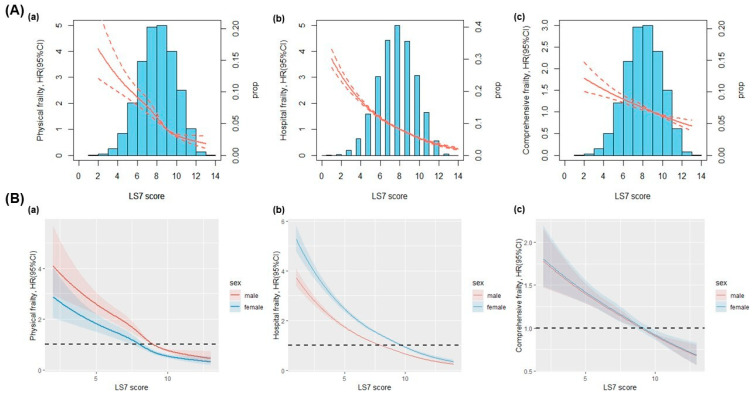
Risk for incident frailty (physical frailty (**a**), hospital frailty (**b**), and comprehensive frailty (**c**)) according to LS7 (LS7 score (**A**), and sex-specific of LS7 score (**B**)) using restricted cubic spline functions in Cox proportional-hazards regression models. The blue bars (histogram) represent the proportion of distribution of each LS7 score. Dotted lines represent the 95% confidence intervals. All models were adjusted for sex, age, race, education, income, alcohol status, diabetes status, and psychosocial health. Physical frailty was additionally adjusted for family history of CVD, stroke status, hypertension status, and CVD status.

Dose–response relationship analyses showed a clear negative association between LS7 score and risk of physical frailty, comprehensive frailty, and hospital frailty (i.e., higher LS7 score was related to lower risk of frailty), with a non-linear relationship (*p* < 0.05) for physical frailty and hospital frailty [Figure 1A(a,b)], and a linear relationship (*p* > 0.05) for comprehensive frailty [Figure 1A(c)]. After the dose–response relationship was stratified by sex, we observed a sex difference in the association with physical frailty [Figure 1B(a)] and hospital frailty [Figure 1B(b)]. In the association with physical frailty, a lower LS7 score was linked to a higher risk of frailty in males than in females, while, in the association with hospital frailty, a lower LS7 score was linked to a higher risk of frailty in females than in males. There was no sex difference in the association with comprehensive frailty [Figure 1B(c)].

### 3.3. Psychosocial Health and Physical Frailty, Hospital Frailty, and Comprehensive Frailty

Poor psychosocial health was associated with an increased risk of frailty; the HRs (95%CI) was 1.87 (1.44–2.43) for physical frailty, 1.46 (1.24–1.71) for comprehensive frailty, and 1.53 (1.44–1.62) for hospital frailty (shown in Table 2 and Table 3). There was a sex difference in the association between social isolation and physical frailty, and the link was only observed in males.

### 3.4. Synergistic Interactions between LS7 Score and Psychosocial Health Status on Risk of Frailty

**Figure 2:**
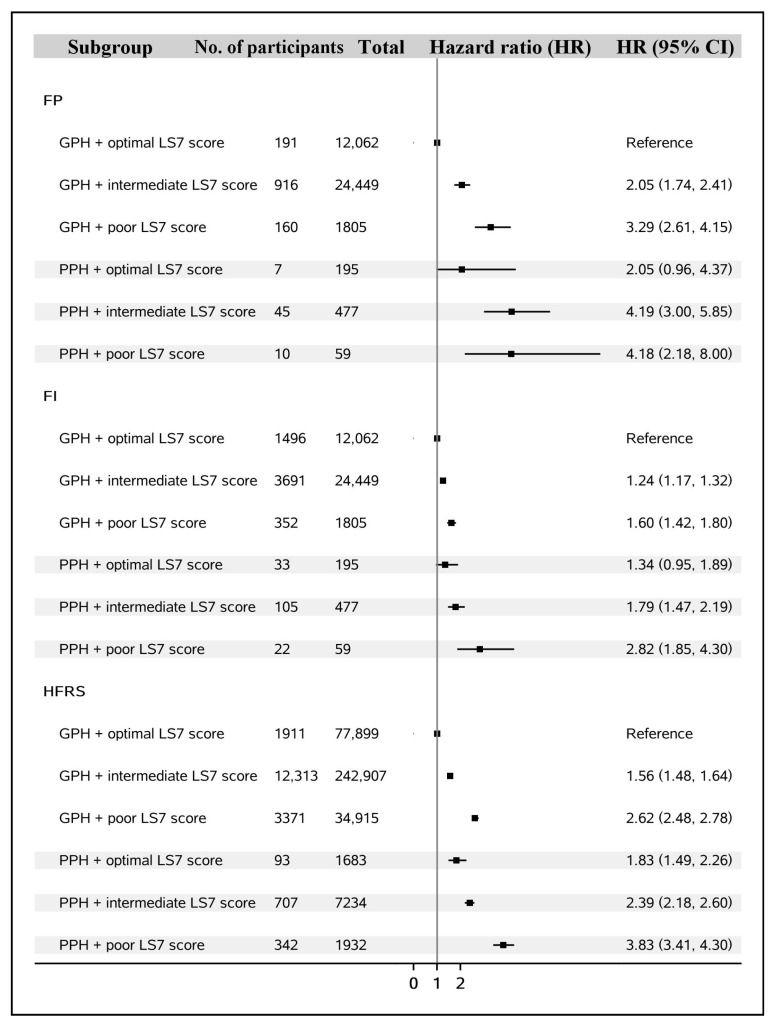
The synergistic effects of LS7 and psychosocial health on frailty. Abbreviations: GPH: good psychosocial health; PPH: poor psychosocial health; HRs: hazard ratios; FP: physical frailty phenotype; FI: frailty index; HFRS: hospital frailty risk score; 95% CI: 95% confidence interval.

We observed synergistic effects of LS7 and psychosocial health on frailty (shown in Figure 2). People who had a poor psychosocial status and poor LS7 score had the highest risk of frailty (physical frailty: HR = 4.18, 95% CI = 2.18–8.00; comprehensive frailty: HR = 2.82, 95% CI = 1.85–4.30; and hospital frailty: HR = 3.83, 95% CI = 3.41–4.30).

## 4. Discussion

In this large population-based prospective cohort study, using three domains of frailty (physical, hospital, and comprehensive), our findings showed that both a better midlife LS7 score and good psychosocial health status were associated with a lower risk of frailty over a 10-year follow-up period. A dose–response relationship was also observed between the LS7 score and frailty, and there was a sex difference between them. We also found a synergistic interaction of LS7 and psychosocial health on frailty. Individuals who had poor psychosocial health and a poor LS7 score were associated with the highest risk of frailty.

### 4.1. LS7 Score and Physical Frailty, Hospital Frailty, and Comprehensive Frailty

Our study extended the limited evidence to date on the association between LS7 and frailty risk. The prospective Nutrition and Cardiovascular Risk in Spain (ENRICA) Study [7] with elderly adults found that a higher LS7 score was associated with a reduced risk of frailty. However, the ENRICA study was conducted in older adults (≥60 years) and only considered the link to physical frailty. In line with Palta’s findings [8], we also observed that a higher LS7 score (i.e., better CVH) in midlife was associated with a lower risk of physical frailty in late life. To the best of our knowledge, no studies have explored the association of LS7 with hospital frailty and comprehensive frailty; our findings demonstrated evidence linking LS7 with hospital frailty and comprehensive frailty.

Consistent with Palta’s findings [8], we found that an optimal BMI and diet were associated with a lower risk of physical frailty. As a complement, we expanded this relationship to comprehensive frailty and hospital frailty. We also found no association between the biometric score of LS7 and physical frailty and comprehensive frailty, congruent with the ENRICA study [7]. Moreover, Boreskie et al. and Ramsay et al. also found no association between physical frailty or comprehensive frailty and biometric score, such as low-density lipoprotein (LDL) [23] and hypertension [24]. However, the biometric score was associated with hospital frailty in our study. One possible explanation is that both high blood pressure and high LDL are two of the most common chronic diseases and are also major risk factors for multiple conditions, leading to multimorbidity [25,26]. Multimorbidity prevalence reaches 55–98% in people older than 65 years [27]. A bidirectional causal relationship between frailty and multimorbidity is presumable [28]. Frailty may predispose persons to the development of multiple chronic diseases, but frailty may also stem from the coexistence of multiple diseases.

Whether there was a sex difference in the association between LS7 and frailty remains unclear. A previous study did not observe a sex difference in the association between LS7 and physical frailty [8]. We observed that a lower LS7 score increased the risk of physical frailty more in males than in females, while a lower LS7 score increased hospital frailty risk more in females than in males. By using physical frailty, Nina Mielke et al. also found that, with unfavorable risk factors, men worsened more often, and those who were already frail died more often than women [29]. Moreover, in men and women over 65 years, studies have found women had a higher overall prevalence of multimorbidity than men [30,31]. These may explain why a lower LS7 score predicted physical frailty better in males, and predicted hospital frailty better in females.

### 4.2. Psychosocial Health and Physical Frailty, Hospital Frailty, and Comprehensive Frailty

Additionally, previous studies yielded inconsistent findings on the association between psychosocial health and frailty. The English Longitudinal Study of Ageing (ELSA) study found no association between social isolation and physical frailty and comprehensive frailty over time [32]. However, this study was subjected to a small sample size and short follow-up period. Another cohort study supported that higher levels of loneliness and social isolation were significantly associated with the risk of worsening physical frailty or pre-frailty in healthy people [10]. Consistent with Jarach’s study, our findings showed that both social isolation and loneliness increased the risk of physical, hospital, and comprehensive frailty.

In addition, in line with the findings of the ELSA study [9], males with social isolation had an increased risk of physical frailty, while no such association was observed in females. One possible explanation is that social isolation (e.g., lack of contact with friends or family) implies less physical activities, and males usually have more physical activity than females [33]. Thus, the reduced physical activities had a greater influence in males than in females.

### 4.3. Synergistic Interactions between LS7 Score and Psychosocial Health Status on Risk of Frailty

Frailty represents a multidimensional state of depleted physiologic and psychosocial reserve [34]. Thus, psychosocial factors might be a concomitant of frailty and may predict the future risk of frailty. We found poor psychosocial factors of social isolation and loneliness in midlife both increased the risk of physical, hospital, and comprehensive frailty, indicating that frailty might be caused not only by physical problems, but also by psychological problems. There was also a synergistic effect of LS7 and psychosocial health on frailty. Our findings imply the necessity to pay attention to risk factors of frailty from a holistic health perspective, i.e., from biological, psychological, and social dimensions, to surveillance and follow-up of the risk factors.

### 4.4. Strength and Limitations

The UK Biobank is a large, prospective, general population cohort with data available on a wide range of potential confounders and health outcomes. As a result, our analyses could broadly analyze the relationship between LS7 score (including lifestyle score and biometric score), psychosocial status, and multi-dimensions of frailty, i.e., physical, hospital, and comprehensive frailty. Frailty represents a multidimensional state of depleted physiologic and psychosocial reserve and clinical vulnerability. Thus, the risk factors of frailty might be also multi-dimensional. We considered the risk factors of frailty based on the bio-psycho-social medical model, and demonstrated a synergistic interaction between the physiological and psycho-social on frailty. Furthermore, the frailty metrics of FP, HFRS, and FI we used in this study have been validated, which enhances the reliability of our findings.

Several limitations need to be acknowledged when interpreting our findings. First, the UK Biobank is not a nationally representative sample, with generally healthier behaviors [35], and less socioeconomically deprived [36] participants than the UK average. Consequently, findings might not be generalizable to other ethnicities or populations. Second, although we adjusted for major confounding factors, residual confounding from unknown or unmeasured factors remains a possibility, such as sleep health [37]. Third, only association and not causation should be inferred because of the observational study design. However, the prospective cohort design provided support for a causal association.

## 5. Conclusions

Based on a bio-psycho-social medical model, we explored the relationships between LS7, psychosocial factors, and multidomain frailties. Our findings showed that a better LS7 score in midlife was associated with a 70%, 60%, and 40% reduced risk of physical frailty, hospital frailty, and comprehensive frailty over 10 years later, respectively. The psychosocial factors of social isolation and loneliness also increased the risk of frailty. There was a synergistic effect of psychosocial status and LS7 on frailty. Individuals who had poor psychosocial health and a poor LS7 score were associated with the highest risk of frailty. Follow-up and surveillance of the LS7 score and psychosocial status is useful for assessing the future risk of frailty, identifying high-risk groups, and developing potential interventions. The present study emphasized the significant role of CVH and psychosocial factors in midlife on multiple frailty dimensions in later life, suggesting maintaining an ideal CVH and psychosocial conditions in early life may help to prevent or delay frailty in later life. Future studies are needed to explore the underlying reasons for the sex difference in the association of CVH with physical frailty and hospital frailty.

## Figures and Tables

**Table 1:** Baseline characteristics of 39,047 participants for assessment of physical frailty and comprehensive frailty, *n* (%).

Characteristics	N	LS7 Score				Psychosocial Health		
		Poor (0–5)	Intermediate (6–9)	Optimal (10–14)	*p* *	Good	Poor	*p* *
No. of participants	39,047	1864	24,926	12,257		38,316	731	
Age (years, mean ± SD)	64.0 ± 7.6	65.6 ± 6.9	65.1 ± 7.3	61.7 ± 7.6	<0.001	64.1 ± 7.6	62.0 ± 7.5	0.506
Sex					<0.001			0.105
Male	19,138	1206 (64.7)	13,495 (54.1)	4437 (36.2)		18,758 (49.0)	380 (52.0)	
Female	19,909	658 (35.3)	11,431 (45.9)	7820 (63.8)		19,558 (51.0)	351 (48.0)	
Race/ethnicity					0.693			
White	37,984	1809 (97.1)	24,259 (97.3)	11,916 (97.2)		37,295 (97.3)	689 (94.2)	<0.001
Non-white	1063	55 (2.9)	667 (2.7)	341 (2.8)		1021 (2.7)	42 (5.8)	
Education level (years)					<0.001			0.011
≤10	13,759	839 (45.0)	9382 (37.7)	3538 (28.9)		13,470 (35.1)	289 (39.5)	
11–12	5197	259 (13.9)	3270 (13.1)	1668 (13.6)		5091 (13.3)	106 (14.5)	
>12	20,091	766 (41.1)	12,274 (49.2)	7051 (57.5)		19,755 (51.6)	336 (46.0)	
Income level (£)					<0.001			<0.001
Less than 18,000	2928	187 (10.0)	1940 (7.8)	801 (6.5)		2792 (7.2)	136 (18.6)	
18,000 to 30,999	7465	421 (22.6)	5045 (20.2)	1999 (16.3)		7267 (19.0)	198 (27.1)	
31,000 to 51,999	11,715	606 (32.5)	7671 (30.8)	3438 (28.1)		11,492 (30.0)	223 (30.5)	
Greater than 52,000	16,939	650 (34.9)	10,270 (41.2)	6019 (49.1)		16,765 (43.8)	174 (23.8)	
Alcohol status					<0.001			<0.001
Never	905	27 (1.5)	530 (2.1)	348 (2.8)		887 (2.3)	18 (2.5)	
Previous	821	56 (3.0)	532 (2.1)	233 (1.9)		791 (2.1)	30 (4.1)	
Current	37,321	1781 (95.5)	23,864 (95.8)	11,676 (95.3)		36,638 (95.6)	683 (93.4)	
Physical Frailty Phenotype					<0.001			<0.001
Non-frail	15,833	502 (26.9)	9575 (38.4)	5756 (47.0)		15,617 (40.8)	216 (29.6)	
Pre-frail	21,885	1192 (64.0)	14,390 (57.7)	6303 (51.4)		21,432 (55.9)	453 (62.0)	
Frail	1329	170 (9.1)	961 (3.9)	198 (1.6)		1267 (3.3)	62 (8.4)	
Frailty Index					<0.001			<0.001
Robust	10,235	300 (16.1)	6090 (24.4)	3845 (31.4)		10,144 (26.4)	91 (12.4)	
Pre-frail	23,113	1190 (63.8)	15,040 (60.4)	6883 (56.2)		22,633 (59.1)	480 (65.7)	
Frail	5699	374 (20.1)	3796 (15.2)	1529 (12.4)		5539 (14.5)	160 (21.9)	

Abbreviations: N, number of participants; SD, standard deviation. * Calculated by using the *t*-test or chi-square test.

**Table 2:** Hazard ratios (HRs) and 95%CIs for physical frailty and comprehensive frailty by LS7 score and psychosocial health factors.

	Physical Frailty				Comprehensive Frailty			
	Events/N	All Participants	Women	Men	Events/N	All Participants	Women	Men
**LS7 score (lifestyle score + biometric score)**								
Poor	170/1864	1.00	1.00	1.00	374/1864	1.00	1.00	1.00
Intermediate	961/24,926	0.64 (0.54, 0.77) *	0.64 (0.49, 0.83) *	0.65 (0.51, 0.82) *	3796/24,926	0.77 (0.69, 0.86) *	0.81 (0.68, 0.96) *	0.75 (0.66, 0.86) *
Optimal	198/12,257	0.31 (0.25, 0.39) *	0.30 (0.22, 0.42) *	0.31 (0.22, 0.44) *	1529/12,257	0.62 (0.55, 0.69) *	0.63 (0.52, 0.75) *	0.62 (0.53, 0.72) *
**Lifestyle score**								
Poor	112/1253	1.00	1.00	1.00	252/1253	1.00	1.00	1.00
Intermediate	1069/27,162	0.57 (0.47, 0.70) *	0.59 (0.44, 0.80) *	0.55 (0.42, 0.72) *	4097/27,162	0.78 (0.69, 0.89) *	0.87 (0.70, 1.08)	0.73 (0.62, 0.85) *
Optimal	148/10,632	0.23 (0.18, 0.30) *	0.26 (0.18, 0.37) *	0.19 (0.13, 0.29) *	1350/10,632	0.65 (0.56, 0.74) *	0.72 (0.58, 0.90) *	0.60 (0.51, 0.72) *
**Smoking status**								
Poor	126/2452	1.00	1.00	1.00	381/2452	1.00	1.00	1.00
Intermediate	503/13,049	0.80 (0.65, 0.97) *	0.62 (0.47, 0.82) *	0.99 (0.74, 1.32)	2083/13,049	1.09 (0.97, 1.21)	1.14 (0.96, 1.35)	1.04 (0.90, 1.20)
Optimal	700/23,546	0.73 (0.60, 0.88) *	0.65 (0.50, 0.84) *	0.79 (0.59, 1.06)	3235/23,546	0.97 (0.87, 1.08)	1.03 (0.87, 1.22)	0.91 (0.79, 1.05)
**BMI**								
Poor	591/7306	1.00	1.00	1.00	1317/7306	1.00	1.00	1.00
Intermediate	495/16,577	0.47 (0.42, 0.54) *	0.46 (0.39, 0.55) *	0.48 (0.40, 0.58) *	2395/16,577	0.83 (0.77, 0.89) *	0.77 (0.70, 0.86) *	0.88 (0.80, 0.97) *
Optimal	243/15,164	0.26 (0.22, 0.31) *	0.24 (0.19, 0.29) *	0.31 (0.24, 0.41) *	1987/15,164	0.75 (0.69, 0.81) *	0.70 (0.63, 0.77) *	0.82 (0.73, 0.91) *
**Diet**								
Poor	125/3399	1.00	1.00	1.00	514/3399	1.00	1.00	1.00
Intermediate	1118/33,080	0.89 (0.74, 1.07)	0.90 (0.69, 1.18)	0.86 (0.66, 1.12)	4808/33,080	0.91 (0.83, 0.99) *	0.94 (0.82, 1.08)	0.88 (0.78, 0.99) *
Optimal	86/2568	0.74 (0.56, 0.98) *	0.68 (0.45, 1.04)	0.76 (0.52, 1.10)	377/2568	0.83 (0.73, 0.95) *	0.90 (0.73, 1.10)	0.79 (0.66, 0.94) *
**Physical activity**								
Poor	222/3572	1.00	1.00	1.00	618/3572	1.00	1.00	1.00
Intermediate	1038/31,669	0.70 (0.61, 0.81) *	0.77 (0.63, 0.95) *	0.64 (0.52, 0.80) *	4559/31,669	0.90 (0.83, 0.98) *	0.91 (0.80, 1.02)	0.89 (0.79, 1.00)
Optimal	69/3806	0.43 (0.33, 0.57) *	0.50 (0.34, 0.74) *	0.39 (0.27, 0.58) *	522/3806	0.90 (0.80, 1.02)	0.89 (0.75, 1.07)	0.91 (0.77, 1.06)
**Biometric score**								
Poor	53/698	1.00	1.00	1.00	125/698	1.00	1.00	1.00
Intermediate	606/14,571	0.95 (0.71, 1.28)	0.99 (0.63, 1.54)	0.95 (0.64, 1.41)	2259/14,571	0.91 (0.76, 1.09)	0.88 (0.66, 1.17)	0.95 (0.75, 1.20)
Optimal	670/23,778	0.89 (0.66, 1.21)	0.87 (0.55, 1.38)	0.92 (0.61, 1.38)	3315/23,778	0.84 (0.70, 1.01)	0.79 (0.60, 1.04)	0.89 (0.71, 1.13)
**Blood pressure**								
Poor	365/9143	1.00	1.00	1.00	1385/9143	1.00	1.00	1.00
Intermediate	802/23,555	1.03 (0.91, 1.18)	0.91 (0.76, 1.09)	1.16 (0.97, 1.40)	3481/23,555	1.02 (0.96, 1.09)	0.98 (0.89, 1.07)	1.07 (0.98, 1.16)
Optimal	162/6349	1.18 (0.96, 1.45)	1.04 (0.81, 1.34)	1.27 (0.85, 1.89)	833/6349	0.98 (0.89, 1.07)	0.95 (0.85, 1.07)	0.94 (0.80, 1.11)
**Cholesterol levels (LDL)**								
Poor	325/9363	1.00	1.00	1.00	1358/9363	1.00	1.00	1.00
Intermediate	737/18,068	1.07 (0.93, 1.22)	0.97 (0.81, 1.16)	1.19 (0.96, 1.47)	2855/18,068	1.07 (1.00, 1.14)	1.04 (0.94, 1.14)	1.09 (0.99, 1.20)
Optimal	267/11,616	0.91 (0.77, 1.07)	0.92 (0.74, 1.13)	0.83 (0.63, 1.11)	1486/11,616	0.94 (0.87, 1.01)	0.92 (0.83, 1.02)	0.93 (0.83, 1.04)
**Glycemic status (HbA1c)**								
Poor	86/780	1.00	1.00	1.00	175/780	1.00	1.00	1.00
Intermediate	255/4356	0.97 (0.74, 1.27)	1.05 (0.68, 1.62)	0.94 (0.67, 1.34)	770/4356	0.84 (0.71, 0.99) *	0.90 (0.68, 1.19)	0.82 (0.67, 1.01)
Optimal	988/33,911	0.85 (0.64, 1.14)	1.06 (0.67, 1.69)	0.70 (0.48, 1.02)	4754/33,911	0.72 (0.62, 0.84) *	0.78 (0.60, 1.02)	0.70 (0.58, 0.85) *
**Psychosocial health status (social isolation + loneliness)**								
Good	1267/38,316	1.00	1.00	1.00	5539/38,316	1.00	1.00	1.00
Poor	62/731	1.87 (1.44, 2.43) *	1.47 (1.01, 2.15) *	2.43 (1.68, 3.50) *	160/731	1.46 (1.24, 1.71) *	1.32 (1.04, 1.67) *	1.59 (1.28, 1.97) *
**Social isolation**								
No	1182/36,193	1.00	1.00	1.00	5192/36,193	1.00	1.00	1.00
Yes	147/2854	1.29 (1.08, 1.54) *	1.10 (0.85, 1.43)	1.48 (1.16, 1.89) *	507/2854	1.19 (1.09, 1.31) *	1.27 (1.11, 1.45) *	1.13 (0.99, 1.28)
**Loneliness**								
No	1013/33,628	1.00	1.00	1.00	4771/33,628	1.00	1.00	1.00
Yes	316/5419	1.67 (1.46, 1.90) *	1.68 (1.42, 1.99) *	1.65 (1.34, 2.00) *	928/5419	1.20 (1.11, 1.28) *	1.18 (1.07, 1.30) *	1.23 (1.11, 1.38) *

All models were adjusted for age, race, education, income, alcohol status, psychosocial health (for LS7 score, and its composition), the lifestyle score (for biometric score), biometric score (for lifestyle score), and LS7 score (for psychosocial health, and its composition). Physical frailty was additionally adjusted for diabetes status, family history of CVD, stroke status, hypertension status, and CVD status. Abbreviations: BMI, body mass index; HbA1c, glycated hemoglobin A1c; LDL, low-density lipoprotein. * *p* < 0.05.

**Table 3:** Hazard ratios (HRs) and 95%CIs for hospital frailty according to LS7 levels and psychosocial health factors ^a^.

	Events/N	All Participants	Women	Men
**LS7 score (lifestyle score + biometric score)**				
Poor	3713/36,847	1.00	1.00	1.00
Intermediate	13,020/250,141	0.60 (0.58, 0.62) *	0.59 (0.55, 0.62) *	0.60 (0.58, 0.63) *
Optimal	2004/79,582	0.39 (0.37, 0.41) *	0.35 (0.32, 0.38) *	0.43 (0.40, 0.46) *
**Lifestyle score**				
Poor	2884/31,907	1.00	1.00	1.00
Intermediate	13,900/270,879	0.64 (0.62, 0.67) *	0.61 (0.57, 0.65) *	0.66 (0.63, 0.70) *
Optimal	1953/63,784	0.43 (0.41, 0.46) *	0.42 (0.38, 0.45) *	0.45 (0.41, 0.49) *
**Smoking status**				
Poor	2971/36,987	1.00	1.00	1.00
Intermediate	7993/129,052	0.76 (0.73, 0.80) *	0.70 (0.66, 0.75) *	0.82 (0.77, 0.87) *
Optimal	7823/200,531	0.58 (0.56, 0.61) *	0.55 (0.52, 0.59) *	0.61 (0.58, 0.65) *
**BMI**				
Poor	6794/88,695	1.00	1.00	1.00
Intermediate	7159/154,743	0.75 (0.72, 0.78) *	0.74 (0.70, 0.78) *	0.76 (0.73, 0.80) *
Optimal	4784/123,132	0.77 (0.74, 0.80) *	0.71 (0.67, 0.75) *	0.84 (0.79, 0.89) *
**Diet**				
Poor	5735/110,620	1.00	1.00	1.00
Intermediate	12,712/249,819	0.94 (0.91, 0.97) *	0.97 (0.92, 1.02)	0.92 (0.88, 0.96) *
Optimal	290/6131	0.69 (0.62, 0.78) *	0.81 (0.68, 0.98) *	0.62 (0.53, 0.72) *
**Physical activity**				
Poor	2952/38,506	1.00	1.00	1.00
Intermediate	13,445/284,383	0.76 (0.73, 0.79) *	0.73 (0.69, 0.78) *	0.78 (0.74, 0.82) *
Optimal	2340/43,681	0.78 (0.73, 0.82) *	0.81 (0.74, 0.88) *	0.76 (0.71, 0.82) *
**Biometric score**				
Poor	1139/10,126	1.00	1.00	1.00
Intermediate	9357/150,371	0.65 (0.61, 0.69) *	0.62 (0.57, 0.68) *	0.67 (0.61, 0.72) *
Optimal	8241/206,073	0.55 (0.52, 0.59) *	0.50 (0.46, 0.55) *	0.59 (0.55, 0.65) *
**Blood pressure**				
Poor	6221/97,863	1.00	1.00	1.00
Intermediate	11,163/217,499	0.93 (0.90, 0.96) *	0.89 (0.85, 0.93) *	0.95 (0.91, 0.99) *
Optimal	1353/51,208	0.74 (0.70, 0.79) *	0.65 (0.60, 0.70) *	0.92 (0.84, 1.01)
**Cholesterol levels (LDL)**				
Poor	3538/89,353	1.00	1.00	1.00
Intermediate	11,043/171,976	1.42 (1.36, 1.47) *	1.32 (1.26, 1.39) *	1.55 (1.46, 1.64) *
Optimal	4156/105,241	1.32 (1.26, 1.38) *	1.16 (1.08, 1.23) *	1.51 (1.41, 1.61) *
**Glycemic status (HbA1c)**				
Poor	2395/14,867	1.00	1.00	1.00
Intermediate	4262/53,509	0.59 (0.56, 0.62) *	0.56 (0.51, 0.60) *	0.62 (0.58, 0.66) *
Optimal	12,080/298,194	0.42 (0.40, 0.44) *	0.41 (0.38, 0.44) *	0.43 (0.40, 0.45) *
**Psychosocial health (social isolation + loneliness)**				
Good	17,595/355,721	1.00	1.00	1.00
Poor	1142/10,849	1.53 (1.44, 1.62) *	1.52 (1.39, 1.66) *	1.54 (1.41, 1.68) *
**Social isolation**				
No	16,046/334,232	1.00	1.00	1.00
Yes	2691/32,338	1.36 (1.31, 1.42) *	1.36 (1.28, 1.45) *	1.37 (1.29, 1.45) *
**Loneliness**				
No	14,082/301,219	1.00	1.00	1.00
Yes	4655/65,351	1.40 (1.35, 1.45) *	1.36 (1.30, 1.43) *	1.45 (1.38, 1.52) *

^a^ Adjusted for sex, age, race, education, income, alcohol status, psychosocial health (for LS7 score, lifestyle score, biometric score, and its composition), the lifestyle score (for biometric score), biometric score (for lifestyle score), and LS7 score (for social isolation, loneliness, and psychosocial health). * *p* < 0.05.

## Data Availability

Researchers can apply to use the UK Biobank resource (https://www.ukbiobank.ac.uk/, accessed on 16 August 2022) and access the data used in this study.

## Supplementary Materials

The following supporting information can be downloaded at: https://www.mdpi.com/article/10.3390/nu15102412/s1, Table S1: UK biobank fields used in the construction of the Life’s Simple 7 score; Table S2: Definition of individual components of the Life’s Simple 7 score in the UK Biobank; Table S3: Definition and UK Biobank fields of the psychosocial health status factors; Table S4: The five frailty criteria for construction of the physical frailty phenotype; Table S5: The frailty items and scoring for construction of the frailty index; Table S6: The ICD-10 codes and assigned weights used to construct the Hospital Frailty Risk Score; Table S7: Baseline characteristics of Hospital Frailty Risk Score. References [38,39,40] are cited in the Supplementary Materials.
