# Supplementary material for: Midlife Life’s Simple 7, Psychosocial Health, and Physical Frailty, Hospital Frailty, and Comprehensive Frailty 10 Years Later
Source: Nutrients. 2023 May 22;15(10):2412. doi: 10.3390/nu15102412 (PMC10223731; doi:10.3390/nu15102412)
Supplement: Supplementary file 1 [file nutrients-15-02412-s001.zip › nutrients-2375368-supplementary.pdf]

Supplementary Materials

Table S1: UK biobank fields used in the construction of the Life's Simple 7 score

Table S2: Definition of individual components of the Life's Simple 7 score in the UK Biobank

Table S3: Definition and UK Biobank fields of the psychosocial health status factors

Table S4: The five frailty criteria for construction of the physical frailty phenotype

Table S5: The frailty items and scoring for construction of the frailty index

Table S6: The ICD-10 codes and assigned weights used to construct the Hospital Frailty Risk Score

Table S7: Baseline characteristics of Hospital Frailty Risk Score

Table S1. UK biobank fields used in the construction of the Life's Simple 7 score

**Life's Simple 7 item**	**UKB fields used**
Smoking status	20116
BMI	21001
Physical activity	884, 904
Diet	1309, 1319, 1289, 1299, 1448, 1438, 1468, 1458, 1329, 1339, 1408, 1418, 1428, 2654, 1349, 1359, 1369, 1379,1389, 3680
Blood pressure	4080, 4079
Cholesterol levels	30780
Glycemic status (HbA1C)	30750

Table S2. Definition of individual components of the Life's Simple 7 score in the UK Biobank

**Life's Simple 7 score**	**Lifestyle scale**				**Biometric scale**		
	**Smoking status**	**BMI**	**Physical activity**	**Diet***	**Blood pressure**	**Cholesterol levels**	**Glycemic status**
**Optimal (score=2)**	Never smoked	<25 kg/m^2	>4 days/week of moderate/vigorous physical activity	>7	SBP<120 mm Hg and DBP< 80 mm Hg untreated	LDL-C <130 mg/dl	HbA1c <5.7%
**Intermediate (score=1)**	Former smoker	25-29.9 kg/m^2	≤4 days/week of moderate/vigorous physical activity	4-7	SBP 120-139 or DBP 80-89 mm Hg OR SBP <120 mm Hg and DBP <80 mm Hg treated	LDL-C 130-159 mg/dl OR LDL-C <130 mg/dl treated	HbA1c 5.7-6.4% OR HbA1c <5.7% treated
**Poor (score=0)**	Current smoker	≥ 30kg/m^2	No moderate/ vigorous physical activity	<4	SBP ≥140 mm Hg or DBP ≥ 90 mm Hg	LDL-C ≥ 160 mg/dl	HbA1c ≥ 6.4%

*Healthy diet score according to Mozaffarian[38] and Said[39] et al.; higher scores indicate adherence to a healthier diet for prevention of cardiovascular disease. BMI, body mass index; SBP, systolic blood pressure; DBP, diastolic blood pressure; LDL-C, low-density lipoprotein cholesterol; HbA1c, glycated hemoglobin.

Table S3. Definition and UK Biobank fields of the psychosocial health status factors

Psychosocial health factors	Touchscreen questionnaire	UKB fields used	Judgment
**Social isolation**	(i) "Including yourself, how many people are living together in your household?" **(1 point if living alone)**	709	**No** (composite score <2); **Yes** (composite score ≥2)
	(ii) "How often do you visit friends or family or have them visit you?" (**1 point if friends/ family visits less than once a month**)	1031	
	(iii) "Which of the following [leisure/social activities] do you engage in once a week or more often?" (**1 point if no activities selected**)	6160	
**Loneliness**	"Do you often feel lonely?	2020	**No; Yes**

Table S4. The five frailty criteria for construction of the physical frailty phenotype

Item	UKB fields	Question/Description	Score
Weight loss	2306	Weight change compared with 1 year ago	Yes, lost weight=1; other=0
Exhaustion	2080	Frequency of tiredness/lethargy in last weeks	More than half the days or nearly every day=1; other=0
Slow walking speed	924	Usual walking pace	Slow=1; other=0
Low physical activity	6164<br>2624<br>1011<br>3637<br>991<br>971	Types of physical activity in last 4 weeks<br>Frequency of heavy DIY in last 4 weeks<br>Frequency of light DIY in last 4 weeks<br>Frequency of other exercises in last 4 weeks<br>Frequency of strenuous sports in last 4 weeks<br>Frequency of walking for pleasure in last weeks	None or light activity with a frequency of once per week or less=1;<br>medium or heavy activity, or light activity more than once per week=0
Low grip strength	46<br>47<br>31<br>21001	Hand grip strength(left)<br>hand grip strength (right)<br>sex<br>BMI	sex and BMI adjusted cutoffs:<br>Men:<br>≤29 kg for BMI ≤24;<br>≤30 kg for BMI 24.1-26;<br>≤30 kg for BMI 26.1-28;<br>≤32 kg for BMI >28<br>Women:<br>≤17 kg for BMI ≤23;<br>≤17.3 kg for BMI 23.1-26;<br>≤18 kg for BMI 26.1-29;<br>≤21 kg for BMI>29

Table adapted from Fried[40] and Hanlon[16], et al. BMI, body mass index.

Table S5. The frailty items and scoring for construction of the frailty index

Type of deficit	ID	Item	UKB Fields	Score
Cancer	1	Any cancer diagnosis*	134, 2453	No=0; Yes=1
	2	Multiple cancers diagnosed	134	No/single=0; Multiple cancer=1
Sensory	3	Glaucoma*	20002 (1277), 6148, 2227	No=0; Yes=1
	4	Cataracts	20002 (1278), 6148, 2227	No=0; Yes=1
	5	Hearing difficulty	2247	No=0; Yes/completely deaf=1
Cranial	6	Migraine*	20002 (1265), 2473	No=0; Yes=1
	7	Mouth/teeth dental problems	6149	None=0; Any=1
Mental wellbeing¶	8	Fatigue: frequency of tiredness/lethargy in last two weeks	2080	Not at all=0; Several days=0.25; More than half=0.5; Nearly every day=1
	9	Self-rated health	2178	Excellent=0; Good=0.25; Fair=0.5; Poor=1
	10	Sleep: experience of sleeplessness/insomnia	1200	Never/rarely=0; Sometimes=0.5; Usually=1
	11	Depressed feelings: frequency in last two weeks	2050	Not at all=0; Several days=0.25; More than half=0.5; Nearly every day=1
	12	Self-described nervous personality	1970	No=0; Yes=1
	13	Severe anxiety/ panic attacks*	20002 (1287), 2473	No=0; Yes=1
	14	Sense of misery	1930	No=0; Yes=1
Infirmity	15	Infirmity: long-standing illness or disability	2188	No=0; Yes=1
	16	Falls in last year	2296	No falls=0; One fall=0.5; More than one=1
	17	Fractures/broken bones in last five years	2463	No=0; Yes=1
Respiratory	18	Breathing: wheeze in last year	2316	No=0; Yes=1
	19	Pneumonia*	20002 (1398), 2473	No=0; Yes=1
	20	Chronic bronchitis/ emphysema*	20002 (1113), 6152	No=0; Yes=1
	21	Asthma*	20002 (1111), 6152	No=0; Yes=1
Pain	22	Chest pain	2335	No=0; Yes=1

	23	Whole-body pain	6159	No=0; Yes=1
	24	Hip pain	6159	No=0; Yes=1
	25	Back pain	6159	No=0; Yes=1
	26	Stomach/abdominal pain	6159	No=0; Yes=1
	27	Knee pain	6159	No=0; Yes=1
	28	Facial pain	6159	No=0; Yes=1
	29	Head and/or neck pain	6159	No=0; Yes=1
	30	Sciatica*	20002 (1476), 2473	No=0; Yes=1
Cardiometabolic	31	Diabetes*	20002 (1220,1223), 2443	No=0; Yes=1
	32	Myocardial infarction*	20002 (1075), 6150	No=0; Yes=1
	33	Angina*	20002 (1074), 6150	No=0; Yes=1
	34	Stroke*	20002 (1081,1583), 6150	No=0; Yes=1
	35	High blood pressure*	20002 (1065), 6150	No=0; Yes=1
	36	Hypothyroidism*	20002 (1226), 2473	No=0; Yes=1
	37	Deep-vein thrombosis*	20002 (1094), 6152	No=0; Yes=1
	38	High cholesterol*	20002 (1473), 6153, 6177	No=0; Yes=1
Musculoskeletal	39	Rheumatoid arthritis*	20002 (1464), 2473	No=0; Yes=1
	40	Osteoarthritis*	20002 (1465), 2473	No=0; Yes=1
	41	Gout*	20002 (1466), 2473	No=0; Yes=1
	42	Osteoporosis*	20002 (1309), 2473	No=0; Yes=1
Immunological	43	Hay fever/allergic rhinitis or eczema*	20002 (1387/1452), 6152	No=0; Yes=1
	44	Psoriasis*	20002 (1453), 2473	No=0; Yes=1
Gastrointestinal	45	Gastric reflux*	20002 (1138), 2473	No=0; Yes=1
	46	Hiatus hernia*	20002 (1474), 2473	No=0; Yes=1
	47	Gall stones*	20002 (1162), 2473	No=0; Yes=1
	48	Diverticulitis*	20002 (1458), 2473	No=0; Yes=1

* Participants reported medically diagnosed conditions for these items. Variables included in frailty index from Williams[18] et al. ¶ We excluded loneliness item because it is also a variable in psychosocial health factors. For items with multiple corresponding data fields, we first coded "Yes" and "No" responses using the self-report items and, including for data field 2473. Additional "Yes" responses were then ascertained from the nurse led interview diagnostic codes (data field 20002). Individuals with missing data for both data fields 2473 and 20002 were coded as missing data. Other criteria regarding the definition of items were shown in Mutz[20], et al.

Table S6. The ICD-10 codes and assigned weights used to construct the Hospital Frailty Risk Score

Num	ICD-10 code	Description	Weight
1	F00	Dementia in Alzheimer's disease	7.1
2	G81	Hemiplegia	4.4
3	G30	Alzheimer's disease	4.0
4	I69	Sequelae of cerebrovascular disease (secondary codes)	3.7
5	R29	Other symptoms and signs involving the nervous and musculoskeletal systems (R29.6Tendency to fall)	3.6
6	N39	Other disorders of urinary system (includes urinary tract infection and urinary in continence)	3.2
7	F05	Delirium, not induced by alcohol and other psychoactive substances	3.2
8	W19	Unspecified fall	3.2
9	S00	Superficial injury of head	3.2
10	R31	Unspecified hematuria	3.0
11	B96	Other bacterial agents as the cause of diseases classified to other chapters (secondary code)	2.9
12	R41	Other symptoms and signs involving cognitive functions and awareness	2.7
13	R26	Abnormalities of gait and mobility	2.6
14	I67	Other cerebrovascular diseases	2.6
15	R56	Convulsions, not elsewhere classified	2.6
16	R40	Somnolence, stupor and coma	2.5
17	T83	Complications of genitourinary prosthetic devices, implants and grafts	2.4
18	S06	Intracranial injury	2.4
19	S42	Fracture of shoulder and upper arm	2.3
20	E87	Other disorders of fluid, electrolyte and acid- base balance	2.3
21	M25	Other joint disorders, not elsewhere classified	2.3
22	E86	Volume depletion	2.3
23	R54	Senility	2.2
24	Z50	Care involving use of rehabilitation procedures	2.1
25	F03	Unspecified dementia	2.1
26	W18	Other fall on same level	2.1
27	Z75	Problems related to medical facilities and other health care	2.0
28	F01	Vascular dementia	2.0
29	S80	Superficial injury of lower leg	2.0
30	L03	Cellulitis	2.0
31	H54	Blindness and low vision	1.9
32	E53	Deficiency of other B group vitamins	1.9
33	Z60	Problems related to social environment	1.8
34	G20	Parkinson's disease	1.8
35	R55	Syncope and collapse	1.8
36	S22	Fracture of rib(s), sternum and thoracic spine	1.8
37	K59	Other functional intestinal disorders	1.8
38	N17	Acute renal failure	1.8

39	L89	Decubitus ulcer	1.7
40	Z22	Carrier of infectious disease	1.7
41	B95	Streptococcus and staphylococcus as the cause of diseases classified to other chapters	1.7
42	L97	Ulcer of lower limb, not elsewhere classified	1.6
43	R44	Other symptoms and signs involving general sensations and perceptions	1.6
44	K26	Duodenal ulcer	1.6
45	I95	Hypotension	1.6
46	N19	Unspecified renal failure	1.6
47	A41	Other septicemia	1.6
48	Z87	Personal history of other diseases and conditions	1.5
49	J96	Respiratory failure, not elsewhere classified	1.5
50	X59	Exposure to unspecified factor	1.5
51	M19	Other arthrosis	1.5
52	G40	Epilepsy	1.5
53	M81	Osteoporosis without pathological fracture	1.4
54	S72	Fracture of femur	1.4
55	S32	Fracture of lumbar spine and pelvis	1.4
56	E16	Other disorders of pancreatic internal secretion	1.4
57	R94	Abnormal results of function studies	1.4
58	N18	Chronic renal failure	1.4
59	R33	Retention of urine	1.3
60	R69	Unknown and unspecified causes of morbidity	1.3
61	N28	Other disorders of kidney and ureter, not elsewhere classified	1.3
62	R32	Unspecified urinary incontinence	1.2
63	G31	Other degenerative diseases of nervous system, not elsewhere classified	1.2
64	Y95	Nosocomial condition	1.2
65	S09	Other and unspecified injuries of head	1.2
66	R45	Symptoms and signs involving emotional state	1.2
67	G45	Transient cerebral ischemic attacks and related syndromes	1.2
68	Z74	Problems related to care-provider dependency	1.1
69	M79	Other soft tissue disorders, not elsewhere classified	1.1
70	W06	Fall involving bed	1.1
71	S01	Open wound of head	1.1
72	A04	Other bacterial intestinal infections	1.1
73	A09	Diarrhea and gastroenteritis of presumed infectious origin	1.1
74	J18	Pneumonia, organism unspecified	1.1
75	J69	Pneumonitis due to solids and liquids	1.0
76	R47	Speech disturbances, not elsewhere classified	1.0
77	E55	Vitamin D deficiency	1.0
78	Z93	Artificial opening status	1.0
79	R02	Gangrene, not elsewhere classified	1.0
80	R63	Symptoms and signs concerning food and fluid intake	0.9
81	H91	Other hearing loss	0.9

82	W10	Fall on and from stairs and steps	0.9
83	W01	Fall on same level from slipping, tripping and stumbling	0.9
84	E05	Thyrotoxicosis [hyperthyroidism]	0.9
85	M41	Scoliosis	0.9
86	R13	Dysphagia	0.8
87	Z99	Dependence on enabling machines and devices	0.8
88	U80	Agent resistant to penicillin and related antibiotics	0.8
89	M80	Osteoporosis with pathological fracture	0.8
90	K92	Other diseases of digestive system	0.8
91	I63	Cerebral Infarction	0.8
92	N20	Calculus of kidney and ureter	0.7
93	F10	Mental and behavioral disorders due to use of alcohol	0.7
94	Y84	Other medical procedures as the cause of abnormal reaction of the patient	0.7
95	R00	Abnormalities of heartbeat	0.7
96	J22	Unspecified acute lower respiratory infection	0.7
97	Z73	Problems related to life-management difficulty	0.6
98	R79	Other abnormal findings of blood chemistry	0.6
99	Z91	Personal history of risk-factors, not elsewhere classified	0.5
100	S51	Open wound of forearm	0.5
101	F32	Depressive episode	0.5
102	M48	Spinal stenosis (secondary code only)	0.5
103	E83	Disorders of mineral metabolism	0.4
104	M15	Polyarthritis	0.4
105	D64	Other anemias	0.4
106	L08	Other local infections of skin and subcutaneous tissue	0.4
107	R11	Nausea and vomiting	0.3
108	K52	Other noninfective gastroenteritis and colitis	0.3
109	R50	Fever of unknown origin	0.1

ICD-10, International Statistical Classification of Diseases and Related Health Problems, Tenth Revision

Table S7. Baseline characteristics of Hospital Frailty Risk Score, *n* (%)

Characteristics	N	LS7 score				Psychosocial health		
		Poor (0-5)	Intermediate (6-9)	Optimal (10-14)	*P**	Good	Poor	*P**
**No. of participants**	366,570	36,847	250,141	79,582		355,721	10,849	
**Age (years), Mean±SD**	69.34±8.09	71.00±7.40	70.11±7.88	66.17±8.21	<0.001	69.36±8.09	68.85±7.95	<0.001
**Sex**					<0.001			<0.001
<Male	168,188	21,102 (57.27)	120,434 (48.15)	26,652 (33.49)		163,385 (45.93)	4803 (44.27)	
Female	198,382	15,745 (42.73)	129,707 (51.85)	52,930 (66.51)		192,336 (54.07)	6046 (55.73)	
**Race/ethnicity**					<0.001			<0.001
White	350,085	35,150 (95.39)	239,120 (95.59)	75,815 (95.27)		340,001 (95.58)	10,084 (92.95)	
Non-white	16,485	1697 (4.61)	11,021 (4.41)	3767 (4.73)		15,720 (4.42)	765 (7.05)	
**Education level (years)**					<0.001			<0.001
<=10	179,259	22,170 (60.17)	126,604 (50.61)	30,485 (38.31)		172,956 (48.62)	6303 (58.10)	
11-12	44,287	4091 (11.10)	29,589 (11.83)	10,607 (13.33)		43,077 (12.11)	1210 (11.15)	
>12	143,024	10,586 (28.73)	93,948 (37.56)	38,490 (48.37)		139,688 (39.27)	3336 (30.75)	
**Income level (£)**					<0.001			<0.001
Less than 18,000	80,086	10,760 (29.20)	56,411 (22.55)	12,915 (16.23)		75,014 (21.09)	5072 (46.75)	
18,000 to 30,999	89,363	9407 (25.53)	62,966 (25.17)	16,990 (21.35)		86,818 (24.41)	2545 (23.46)	
31,000 to 51,999	96,023	8750 (23.75)	65,447 (26.16)	21,826 (27.43)		94,225 (26.49)	1798 (16.57)	
Greater than 52,000	101,098	7930 (21.52)	65,317 (26.11)	27,851 (35.00)		99,664 (28.02)	1434 (13.22)	
**Alcohol status**					<0.001			<0.001
Never	14,072	1270 (3.45)	9414 (3.76)	3388 (4.26)		13,406 (3.77)	666 (6.14)	
Previous	12,344	1822 (4.94)	8226 (3.29)	2296 (2.89)		11,392 (3.20)	952 (8.78)	
Current	340,154	33,755 (91.61)	232,501 (92.95)	73,898 (92.86)		330,923 (93.03)	9231 (85.09)	
**Hospital Frailty Risk Score**					<0.001			<0.001
Low	289,190	24,931 (67.66)	195,523 (78.17)	68,736 (86.37)		281,803 (79.22)	7387 (68.09)	
Intermediate	58,643	8203 (22.26)	41,598 (16.63)	8842 (11.11)		56,323 (15.83)	2320 (21.38)	
High	18,737	3713 (10.08)	13,020 (5.21)	2004 (2.52)		17,595 (4.95)	1142 (10.53)	

Abbreviations: N, number of participants; SD, standard deviation. * Calculated by using the t-test or chi-square test.
